# P-2152. *Strongyloides stercoralis* Seroprevalence among Solid Organ Transplant Candidates and Recipients in a Multicenter Hospital System in Arizona

**DOI:** 10.1093/ofid/ofae631.2306

**Published:** 2025-01-29

**Authors:** Lucia A Luna Wong, Sanjeeva Weerasinghe, Catherine Anania, Skyler Trieu, Mohanad Al-Obaidi, Tirdad Zangeneh

**Affiliations:** University of Washington Infectious Diseases Fellowship, Seattle, Washington; University of Arizona Department of Medicine/Banner University Medical Center - Tucson, Tucson, Arizona; University of Arizona Department of Medicine/ Banner University Medical Center- Tucson, Tucson, Arizona; University of Arizona Department of Medicine/ Banner University Medical Center- Tucson, Tucson, Arizona; University of Arizona Department of Medicine, Division of Infectious Diseases, Tucson, Arizona; University of Arizona, Tucson, Arizona

## Abstract

**Background:**

Solid organ transplant (SOT) recipients have a higher risk of Strongyloidiasis associated complications. Universal screening for *Strongyloides* among SOT candidates and recipients is not well defined in the Southwest United States. We assessed our center’s seropositivity rate for pre- and post-SOT using *Strongyloides* Ab (strAb) test.

**Methods:**

We conducted a multicenter, IRB-approved retrospective study. Data were collected from January 2019 to March 2024 for patients ≥18 years old before and after SOT. Most of the testing at our center was performed utilizing the *Strongyloides* ELISA IgG test kit, Gold Standard Diagnostics, Davis, CA, USA.

**Results:**

There were 387 patients with a median age of 61 (range 24-85), the majority being white non-Hispanics, 221 (57%) and 241 (62%) males. There were 152 pre-SOT tests performed: kidney (61), liver (54), lung (17), and other (20), in contrast to the 292 post-SOT: kidney (153), liver (45), lung (41), heart (31), and others (22). The majority (248/387) of patients reported historical travel, with 154 (62%) to *Strongyloides* endemic regions. There were 269 pre-SOT serology, mostly (85%) positive for Epstein Barr virus (EBV), followed by cytomegalovirus (CMV) with 70%, 5% hepatitis C. There was one case of a patient with positive HTLV-I/II serology (negative by confirmatory testing). Of the 283 patients, 24% reported being born outside the US. Of the171 reported gastrointestinal symptoms at the time of strAb testing, 94% were post-SOT. There were 20/387 (5.2%) positive strAb, with 5.3% in pre-SOT and 4.1% in post-SOT. There were 3 cases with negative pre-SOT that seroconverted after transplantation. All of the 93 patients who underwent microscopic stool examinations tested negative. 17/20 patients with a positive strAb received treatment with ivermectin. There was one case of Hyperinfection Syndrome. There was no statistical association between demographics and aforementioned risks, and seropositivity.

**Conclusion:**

The *Strongyloides* seropositivity rate in our cohort is comparable to other reports. No associated risk factor was identified. Therefore, universal screening may be needed in similar transplant cohorts, as undetected cases can have devastating outcomes.

**Disclosures:**

Tirdad Zangeneh, DO, AiCuris.: Grant/Research Support

